# René Dubos, tuberculosis, and the “ecological facets of virulence”

**DOI:** 10.1007/s40656-017-0142-5

**Published:** 2017-07-04

**Authors:** Mark Honigsbaum

**Affiliations:** School of History, Queen Mary University of London, Mile End Road, London, E1 4NS UK

**Keywords:** Ecology, Virulence, Balance, Bacterial adaptation, Tuberculosis, Antibiotics

## Abstract

Reflecting on his scientific career toward the end of his life, the French-educated medical researcher René Dubos presented his flowering as an ecological thinker as a story of linear progression—the inevitable product of the intellectual seeds planted in his youth. But how much store should we set by Dubos’s account of his ecological journey? Resisting retrospective biographical readings, this paper seeks to relate the development of Dubos’s ecological ideas to his experimental practices and his career as a laboratory researcher. In particular, I focus on Dubos’s studies of tuberculosis at the Rockefeller Institute in the period 1944–1956—studies which began with an inquiry into the tubercle bacillus and the physiochemical determinants of virulence, but which soon encompassed a wider investigation of the influence of environmental forces and host–parasite interactions on susceptibility and resistance to infection in animal models. At the same time, through a close reading of Dubos’s scientific papers and correspondence, I show how he both drew on and distinguished his ecological ideas from those of other medical researchers such as Theobald Smith, Frank Macfarlane Burnet, and Frank Fenner. However, whereas Burnet and Fenner tended to view ecological interactions at the level of populations, Dubos focused on the interface of hosts and parasites in the physiological environments of individuals. The result was that although Dubos never fully engaged with the science of ecology, he was able to incorporate ecological ideas into his thought and practices, and relate them to his holistic views on health and the natural harmony of man and his environment.

In January 1950, René Dubos prepared to give a lecture on tuberculosis to the Society of American Bacteriologists in New York. In the talk, the French-educated Rockefeller researcher highlighted what he considered the most striking aspect of the disease: namely, the sequestration of virulent tubercle bacilli in the tissue of recovered patients. “In most other infectious diseases,” Dubos noted, “recovery is usually accompanied by elimination of the infective agent,” but this was not the case with tuberculosis and its serpentine, cord-like bacilli.^1^ Instead, after an attack of primary pulmonary TB, patients could harbor virulent bacilli, sequestered in caseating granulomas, putting them at risk of a recrudescence of disease should their resistance ebb. While vaccination with cultures of Bacillus Calmette–Guérin (BCG) offered a high degree of protection, Dubos worried that such protection could never be complete as long as the components of tubercle bacilli that regulated immunity remained mysterious.^2^ Moreover, using new rapid dispersed culture methods for mycobacteria, Dubos and his former laboratory assistant, the Australian microbiologist, Frank Fenner, had recently established that the BCG vaccine was unstable and comprised mixed populations of virulent and avirulent bacteria, making it potentially hazardous (Dubos et al. 1950a). That was why the work being undertaken at his laboratory at the Rockefeller Institute for Medical Research in New York was so important, he asserted. “By comparing the structure and behavior of the virulent and avirulent forms [of bacilli] we hope to recognize the component or property which endows the virulent form with the ability to produce disease.”^3^ In this way, Dubos explained, he hoped to produce a safer and more reliable vaccine—one that might confer lifelong protection without the risk of a return to virulence.

Although within a few years Dubos would abandon his search for a tuberculosis vaccine, concentrating instead on procedures for the standardization of BCG, at the time his studies were hailed as a major breakthrough in the understanding of the pathogenesis of tuberculosis, leading to the award of the Trudeau Society Medal in 1951. More recently, Dubos’s tuberculosis studies have been seen as a key moment in the evolution of his ecological thought, one that, in the words of his biographer and former research assistant, Carol Moberg, opened up the “ecological facets of virulence” (Moberg 2005, p. 82). At first glance, this reading seems to be supported by Dubos’s pronouncements from the period. For instance, giving the O.T. Avery Lecture at the Society of American Bacteriologists in 1948, Dubos had lamented:

…Little is known of the mechanisms by which tubercle bacilli become established in a new host, and cause disease, or of the processes used by the infected host to overcome the infection. In other words, we know much of the ecological aspects of host-parasite relationships in tuberculosis, hardly anything of the means used by the bacillus to behave as a parasite (Dubos 1948, p. 179).

Three decades later, reflecting on the professional choices and ideas that had shaped his scientific career, Dubos claimed that though he had never taken a course in ecology and had “few occasions to use this word until the 1960s,” ever since 1924, when he had embarked on a professional career as an experimental biologist, he had “always looked at things from an ecological point of view by placing most emphasis *not* on the living things themselves but rather on their interrelationships and on their interplay with surroundings and events.”^4^ In so doing, Dubos presented his flowering as an ecological thinker as the inevitable product of the intellectual seeds planted in his youth when, as a 23-year-old editor working in Rome, he had chanced on an article by the Russian soil microbiologist Sergei Winogradsky and had become “entranced” by the idea that even the smallest living organisms were influenced by environmental conditions, in this case, the chemical composition of soil (Dubos 1974, p. 703). It was this insight that Dubos later claimed had led to his discovery in 1932, together with Oswald Avery, of a soil enzyme (SIII) that decomposed the polysaccharide capsule of pneumococcus, the major cause of lobar pneumonia, and his isolation in 1939 of gramicidin and tyrothricin, the first antibacterial agents. And it was this that in turn had led him to emphasize the relationship between health, disease and the environment in his popular writings and to decry short-term technological fixes, including efforts to eradicate specific diseases, that he feared might upset the delicate ecological equilibrium between humans and microbes.

Rather than accepting Dubos’s account of the evolution of his ecological thought at face value, this paper takes a different approach. Resisting retrospective readings, I argue that Dubos’s and Moberg’s accounts are incomplete and tend to ignore the influence on Dubos’s ecological thinking of the virologist Frank Macfarlane Burnet. Although in interviews towards the end of his life Dubos traced the key shift in his ecological thinking to the late 1930s and early 1940s, a close analysis of his published writings shows that the 1948 Avery lecture was the first time Dubos used ecological terminology. This was 5 years after Dubos had recommended Burnet as the Dunham Lecturer at Harvard University and 8 years after the publication of Burnet’s influential book, *Biological Aspects of Infectious Disease*, which had popularised the Australian researcher’s “ecological point of view” (Burnet 1940, Chapter 1, pp. 1–125). Moreover, the shift in Dubos’s language coincided with the arrival at his laboratory of Burnet’s protégé, Frank Fenner, a microbiologist with experience in the tropics who would go on to conduct a ground-breaking biological study in Australia of the rabbit virus, myxomatosis (Anderson 2017).

Dubos greatly admired Fenner’s studies of myxomatosis, not least because he regarded it as a demonstration of the way that host–parasite interactions under natural conditions favoured equilibrium states and selected for pathogens of lowered virulence—a conclusion that mirrored his laboratory studies of the metabolic and environmental factors that regulated the susceptibility of germ-free laboratory mice to virulent and avirulent tubercle bacilli. Yet though Dubos praised Fenner’s study, he was curiously reluctant to praise Burnet in similar terms or acknowledge the future Australian Nobel Prize winner’s influence on his thought.^5^ This was despite the fact that in his Dunham lectures Burnet had placed virulence at the centre of his epidemiological and ecological analysis, arguing that it was only by exploring the factors governing variations in the virulence of viruses and bacteria that scientists could hope to understand the natural history of infectious disease.^6^ Instead, Dubos would claim that a far greater influence during his time at Harvard had been his interactions with bacteriologists in the laboratory established by Hans Zinsser, such as the virologist John Enders, and his engagement with the writings of Louis Pasteur, Claude Bernard and the American veterinary pathologist, Theobald Smith. It was these thinkers, he asserted, that had prompted his turn to a broader, bio-ecological sensibility.^7^ In this respect Dubos conforms to the pattern of other pioneers of disease ecology, each of whom, according to Anderson, “tended to represent himself … as the sole author of the idea, and rarely cited others, even those linked by education and friendship.” Nevertheless, Anderson suggests that in their training and career paths, Dubos, like Burnet and Smith, “structured an intricate network of influence, counsel and criticism” and, despite their different research interests and professional loyalties, forged “a shared conceptual framework… within infectious disease research” (Anderson 2004, p. 41).

This paper builds on Anderson’s insights. At the same time, through a close reading of Dubos’s scientific papers, books, interviews, and correspondence with other disease researchers, I trace his recourse to ecological terminology and his employment of biological and ecological metaphors. In particular, I concentrate on the middle part of Dubos’s career in the 1940s and 1950s. The first section focuses on the period 1940 to 1944 when Dubos abandoned antibiotics research and left the Rockefeller Institute to become George Fabyean Professor of Comparative Pathology and Tropical Medicine at the Harvard School of Public Health. Dubos’s move was precipitated by the death from tuberculosis in 1942 of his first wife, Marie Louise Bonnet, and coincided with one of his most fertile intellectual periods—a period that resulted in the publication of *The Bacterial Cell*, one of the first books to challenge then notions of bacterial fixity and to call for the incorporation of biological insights into medical research, and also saw him begin his revisionist biography of Pasteur (Dubos 1945, 1950). It was at Harvard that Dubos was also exposed to Burnet’s ecological ideas and had a chance to discuss their mutual research interests. However, through a close reading of Dubos’s scientific papers and publications, I show that Dubos looked to Smith, rather than to Burnet, for an intellectual antecedent for his ecological ideas. In this way, Dubos was able to associate himself with a leading figure in American microbiology while marking the departure of his own biological and ecological thought.

The second section focuses on the period from 1944 to 1956 when Dubos returned to the Rockefeller Institute and launched his experimental studies of tuberculosis. It was in this period that Dubos used novel media to encourage the growth of homogenous tuberculosis bacilli in order to study virulence at a physiochemical level, analysed the BCG vaccine with Fenner, and developed a research program looking at susceptibility and resistance to disease in animal models. However, Dubos was fully aware that his laboratory studies were an approximation of nature and that in order to understand the phenomenon of latent tuberculosis infections and the persistence of the parasite in the environment it was necessary to pay attention to the social conditions governing immunity to tuberculosis in human communities, a theme he explored in *The White Plague*, his historical study of tuberculosis co-written with his second wife, Jean Porter Dubos (Dubos and Dubos 1987). It was also in this period that Dubos became fascinated with Fenner’s study of mxyomatosis as evidence of how under natural conditions long associations between parasites and hosts favoured pathogens of lowered virulence (Anderson 2017).

In the third and final section, I turn to Dubos’s book *Biochemical Determinants of Microbial Diseases* to show how he sought to distinguish his ecological ideas from those of other thinkers (Dubos 1954). In particular, I show how while praising Smith’s contributions, Dubos explicitly contrasted his approach to tuberculosis with Smith’s ideas on equilibrium states. Instead, drawing on Bernard’s idea of the *milieu interieur* and Winogradsky’s studies of the ecology of soil microbes, Dubos increasingly stressed physiochemical factors and the importance of the environment of the host to the regulation of virulence and resistance to infection in animal models.^8^ In conclusion, I argue that while Dubos’s interactions with Burnet and Fenner gave him a deeper appreciation of the role of natural selection in variations in virulence observed in the field, as opposed to the artificial environment of the laboratory, he never fully embraced their model of disease ecology. Instead, for Dubos, ecology served primarily as a metaphor, one that enabled him to connect his scientific work to his broader humanistic concerns and popularise his ideas about the natural harmony of man and the environment.

## From antibiotics to bacterial adaptation

In 1939 René Dubos made a dramatic announcement at New York’s Waldorf Astoria hotel. The occasion was the Third International Microbiology Conference and Dubos had just made a breakthrough that would revolutionize medicine. Holding up a tiny bottle containing five hundred grams of a new antibacterial substance called tyrothricin, Dubos revealed that the vial contained enough gray powder to protect “five trillion mice” against pneumococcal and streptococcal infections. Although tyrothricin had yet to be tested on humans and would later be shown to cause hemolysis when taken into the body, *The New York Time*s immediately grasped the import of Dubos’s announcement, declaring that his discovery “opens up a vast field in the search for chemical agents for fighting bacterial enemies.”^9^

Dubos’s breakthrough while working in Avery’s laboratory at the Rockefeller Institute for Medical Research marked the launch of the antibiotics era proper (Moberg and Cohn 1990; Podolsky 2015). Within months, Dubos’s isolation of tyrothricin had stimulated Howard Florey and Ernst Chain to look further into penicillin and had persuaded Dubos’s former mentor, the Russian-born biochemist Selman Waksman, to undertake a similar search for antibacterial agents using the same soil enrichment techniques, one that would result in his isolation of streptomycin 4 years later (Schatz et al. 1944). Little wonder then that Dubos’s discovery established his scientific reputation, earning him the 1948 Lasker Award and election to the U.S. National Academy of Sciences—no small feat for an agriculturally trained Frenchman without a medical degree. Yet at the very moment of his triumph, Dubos abandoned antibiotics research and by 1942 had quit the Rockefeller for Harvard (Honigsbaum 2016a). Why?

Reflecting on his career in 1981, Dubos would claim that once the basic methods of the search for antibacterial agents had been worked out he ceased to find antibiotics research “intellectually challenging”.

I had become increasingly interested in the mechanisms of disease causation and evolution. My primary scientific interest had become the influence of environmental forces on the susceptibility and resistance of animals and human beings to infection and other forms of stress.^10^

Unfortunately, prior to 1970 Dubos’s practice was to discard his laboratory notebooks, correspondence, and personal papers, so we have no way of comparing his explanation with his thinking at the time.^11^ However, his scientific publications from the period show little interest in the pathology and evolution of infectious disease, much less with the influence of environment at a macro level. Instead, at this stage, Dubos’s focus was very much on the bacterial cell and interactions at a micro level, interactions that triggered his interest in immunochemical processes and adaptive biological responses more generally. This is the more likely intellectual epiphany that prompted Dubos to abandon antibiotics research.

Dubos’s key insight was that bacteria only secreted the SIII enzyme as a sort of emergency measure when they were deprived of other sources of energy for growth. As he put it in a paper published the year after his announcement at the Waldorf Hotel:

The production of adaptive enzymes is a striking example, fairly well defined in biochemical terms, of adaptive responses of the living cell to changes in the environment. A consideration of this phenomenon brings the bacteriologist back into the main channels of biological thought, to the biological problem *par excellence*, the problem of adaptation (Dubos 1940).^12^

This idea, that the bacterial cell could not be understood in isolation from its in vivo environment, would become a key theme of *The Bacterial Cell*, the book that emerged from Dubos’s lectures at the Lowell Institute in Boston in 1944 (Dubos 1945, p. 10).^13^ More significantly, in the light of the subsequent trajectory of Dubos’s biological and ecological thought, it would also lead him to challenge one of the central tenet’s of germ theory: namely, the idea that virulence is a property of microbes alone. While hunting for the SIII enzyme, Dubos had discovered that, although the polysaccharide capsule protected the bacteria from phagocytosis, it was not the only factor governing virulence. Animals with lowered resistance could still develop pneumococcal disease even when the enzyme had made the bacteria vulnerable to attack. In reality, Dubos argued, virulence could not be reduced to the invading microbe, but was a complex phenomenon made up of several factors, including the germ’s ability to invade the host, to survive the host’s defence mechanisms, to multiply within the in vivo environment, and to produce a toxic effect. If any of these factors was blocked then the pathological signs associated with virulence would not appear. “Virulence,” Dubos opined in *The Bacterial Cell*, “is not a permanent, intrinsic property of a given species.”

It expresses only the ability of a given strain of the infective agent, in a certain growth phase, to produce a pathological state in a particular host, when introduced into that host under well-defined conditions. This definition restores to the word virulence much of its earlier meaning; it refers to the disease and to the host-parasite relationship, rather than to some unique attribute of the microorganism. (Dubos 1945, p. 193).

Dubos’s employment of the phrase “host–parasite” clearly reflects the influence on his thought of Theobald Smith. As early as 1903, in an address to the Society of American Bacteriologists, Smith had declared that disease phenomena resided in neither the parasite nor the host but “in the delicate equilibrium between the two which is maintained in various intricate ways” (Smith 1903, p. 233). Expanding this insight in a paper in *Science* the following year, Smith looked to shifts in virulence and evolutionary forces to explain the transmission of a parasite from one type of host to another.

….There will be a selection in favour of those varieties which vegetate once they escape. The surviving varieties would gradually lose their highly virulent invasive qualities and adapt themselves more particularly surrounding invasion and escape. That some such process of selection has been going on in the past seems the simplest explanation of the relatively low mortality of infectious diseases (Smith 1904, p. 825).

Calling his theory “the law of declining virulence,” Smith maintained that as a general rule parasites evolved to a state of “peaceful co-existence” with their hosts so as to favour avirulent, subclinical infections (Smith 1904, pp. 817, 827). As Méthot has shown, Smith’s ideas were hugely influential in bacteriological circles and following the publication of his 1934 book, *Parasitism and Disease*, enjoyed wide currency, particularly in the United States where his ideas were taken up by Zinsser and other leading American microbiologists (Smith 1934). More pertinently for present purposes, Méthot argues that Smith’s theory can also be seen as “an attempt to come to terms with the ecological dynamics of host parasite relations and with the existence of diseases as biological phenomena” (Méthot 2012, p. 594). No doubt it was this aspect that also made Smith’s ideas appealing to Dubos. However, whereas other proto-disease ecologists such as Karl Meyer, the head of the Hooper Foundation in San Francisco, visited Smith regularly at the Department of Animal Pathology in Princeton, Dubos appears to have had little direct contact with him (Honigsbaum 2016b). Instead, he appears to have become acquainted with his ideas largely through reading *Parasitism and Disease* (Moberg 2005, p. 77).

Although *The Bacterial Cell* drew on more than a decade of research at the Rockefeller Institute, Dubos wrote the manuscript at Harvard, an institution he found more conducive to reflection on the philosophical aspects of science, a pursuit that, as he informed an interviewer in 1957, would have been “considered flippant” at the Rockefeller (Benison 1957 IV, p. 568). In explaining the shift in Dubos’ thinking that occurred in this period, Dubos’s biographers usually point to the death of Marie Louise shortly before he took up his Harvard appointment. Shattered by his wife’s death, Dubos vowed to make it his business to “try to unravel the mechanisms of immunological resistance to tuberculosis” and “those physiological characteristics of an individual which make him resistant to tuberculosis one day and highly susceptible another day” (Benison 1957 V, pp. 136–137). However, although Dubos hoped that his new position at Harvard would allow unfettered time for tuberculosis research, his plans were disrupted by the war and administrative duties. As chair of the department, Dubos was burdened with paperwork and tasked with assisting the U.S. government’s biological weapons programme, meaning that any spare time was devoted to the production of Shiga bacillus toxin and the search for a vaccine against bacillary dysentery. Unable to work on tuberculosis, Dubos used his time at Harvard to read up on the history of the disease. His historical reflections were prompted by reports reaching him from army camps of outbreaks of streptococcal and meningococcal disease and the insight that tuberculosis was “not a problem in those communities in which people had come to live… in equilibrium with their environment.” Instead, Dubos “became convinced that the all important factor in tuberculosis disease was…the type of stress and strain which modified the resistance of the individual to infection or modified, for that matter, the resistance of an entire society to infection” (Benison 1957 IV, pp. 561–562). This was the beginning of Dubos’s interest in the physiological mechanisms of the host and the realization that ideas of immune balance offered another way of considering shifts in host–parasite interactions from virulence to avirulence and back again.

Another important influence on Dubos’s thinking in this period were his conversations with his Harvard colleagues. Dubos arrived in Boston too late to meet Zinsser (1878–1940), the former head of Harvard’s bacteriology department, who had died of leukemia in 1940. However, he enjoyed fruitful discussions with Enders (1897–1985). A former English graduate and philologist, Enders had joined Zinsser’s laboratory in 1924 at the age of 30 and, after earning his doctorate, had become Zinsser’s assistant and confidante, so much so that by 1939 he was credited as a co-author of the fifth edition of Zinsser’s textbook on the principles of immunology. Determined to find a way to study viruses in the laboratory, in 1948 Enders, and his colleagues at Boston Children’s Hospital, hit on a method of growing polio virus by inoculating it into material from aborted embryos and fetuses (Weller and Robbins 1981). Until 1948, polio had stubbornly resisted cultivation in cell cultures and, with the exception of yellow fever, virologists had only been able to cultivate other viruses sporadically and with great difficulty. As Thomas Rivers, the director of the Rockefeller Institute and America’s leading virologist remarked, Enders’ discovery, was “like hearing a cannon go off” and cleared the way for scientists to grow other viruses in fetal tissue and develop new vaccines in the years ahead (Benison 1967, p. 446).

Dubos does not say whether he discussed the cultivation of polio with Enders, mentioning only that he was impressed by Enders’s ability to pepper their scientific discussions with classical allusions (Benison 1957 IV, p. 570). However, Dubos was familiar with the textbook on immunology that Zinsser and Enders had co-authored with their colleague LeRoy Fothergill, and it is likely that one of the subjects would have been the nature of immunity and virulence. In the 1931 edition of his textbook, Zinsser had defined virulence as a germ’s “power to invade” (Zinsser 1931, p. 6). At the same time, he recognised that infection could be modified by “secondary factors,” including the number or dose of infecting bacteria, their pathway into the body, and the “environmental conditions” pertaining in host tissue, and cited Smith’s view that long-term associations between hosts and parasites tended to favor commensal states with pathogens of lowered virulence (pp. 3, 17). In particular, Zinsser highlighted the phenomenon of latent infections, pointing out how in the case of tuberculosis and syphilis, infectious bacteria could lie dormant in tissue without giving rise to disease, “yet, at a given moment, often without apparent cause, a characteristic inflammatory process may be ignited” (p. 26). By 1934, in the revised edition of the textbook edited with Enders and Forthergill, Zinsser was ready to abandon his former conception of virulence, arguing that it could no longer be regarded as equivalent to invasiveness as there were “many examples of virulent bacteria that do not ordinarily have the ability to invade tissues and vice versa.” Instead, he advocated a more explicitly biological conception of disease as the interaction of “two living entities—the infectious agent and the host” (Zinsser et al. 1939, pp. 1–5). In this way, Zinsser followed in the tradition established by Smith of thinking about host/parasite interactions in terms of balance or equilibrium. But while, 20 years later, Dubos would acknowledge that Zinsser and Enders’s textbook had been one of his “Bibles” at Harvard, he claimed that the limitations of their immunological conception had already been apparent to him in the 1940s. This was because it was unable to account for certain disease phenomena, such as why resistance to tuberculosis frequently broke down under conditions of “psychological misery” or why, despite possessing neutralizing antibodies to the herpes virus, Dubos’s lips “blossom[ed] with herpes blisters” every time he visited Washington.^14^

Though in interviews and his private papers and published writings, Dubos rarely cites Burnet, the French microbiologist also could not have failed to take note of the ideas outlined by the Australian medical researcher in his Dunham Lectures. Although there is no evidence that Burnet and Dubos corresponded on scientific matters or enjoyed regular intellectual exchanges, Dubos was a member of the Harvard committee that selected the Dunham Lecturer and his recommendation had been key to Burnet’s invitation.^15^ Moreover, on arriving in Boston on 4 January 1944 after a tour of leading American bacteriological laboratories that had begun at the Hooper Foundation in San Francisco 3 weeks earlier, Burnet made directly for Dubos’s office where, according to Burnet’s travel diary, they swapped notes on “evolution by emergence of persisting qualities… [and] mutation by loss theories for salmonellas, viruses and autotrophic bacteria.”^16^ In other words, they discussed the phenomenon of bacterial variation and evolutionary adaptations.

It was a theme that Burnet took up in his Dunham Lectures delivered over 3 days in January 1944. Burnet titled the series, “Some Virus Diseases of Man: Evolutionary and Ecological Considerations” (Burnet 1944). In the first lecture, Burnet presented an overview of the origins of infectious disease, before proceeding in succeeding lectures to more detailed discussions of herpes, poliomyelitis, and influenza. The lectures touched on several themes Burnet had first explored in *Biological Aspects* 4 years earlier, but now expanded and rendered in more technical language for a medical audience. The lectures also majored on virulence. Announcing his intention to broaden the “scope of ecology” so as to take in “the long-term historical aspects of the interaction between organism and environment, i.e. its evolution,” Burnet argued that what the clinician or epidemiologist labelled disease was the result of variations in virulence due to one or a combination of three factors: the emergence of new virulent parasitic variants as a result of spontaneous genetic mutations; changes in the environment or in the habits of a host species; and the opportunity for the transfer of a parasite to a new host species. “Any or all of these may come into play to modify the type of disease toward greater or lesser virulence, to change its standard host or lead to its extinction” (Burnet 1944, pp. 2–3). The problem was that, in practice, virulence was “extraordinarily difficult to define and in every actual instance requir[ed] careful analysis of the factors involved” (p. 7). Later, reworking these ideas for his book *Virus as Organism*, Burnet would place equal emphasis on random genetic mutations and the accidental transfer of saprophytic parasites to new hosts to which they had not had time to adapt. In the short term, he pointed out, such parasites might prove highly pathogenic to the host organism. However, Burnet argued that in the long term natural selection favoured commensal states characterised by low virulence as “an acutely fatal infection is… disadvantageous for the survival of the parasite”—a positioned that echoed Smith (Burnet 1945, pp. 25–26).^17^ Citing Meyer’s work on psittacosis, Burnet also discussed the phenomenon of latent infections, arguing that such states reflected an advanced stage of parasitism or a “balanced interaction” between two species in which parasites persisted without causing excessive harm to their animal hosts, the highest state of all being what Burnet called the “ecological climax condition” (Burnet 1945, pp. 28, 31).

Burnet borrowed this concept from the American plant ecologist, Frederick Clements (1874–1945) who had used it to explain how plants populate landscapes. In Clements’s dynamic ecology, the climax community designated the final stage of a series of vegetative successions at which a plant community could perpetuate itself until an external event, such as sudden change in climate, disturbed the equilibrium, after which the plant community would inevitably progress to the same climax state again (Worster 1994, pp. 205–221). Another key influence on Burnet’s scientific approach to ecology was his engagement with the ideas of Thomas Huxley’s former student, the Oxford animal ecologist Charles Elton. In particular, Anderson’s cites the influence on Burnet of Elton’s 1927 book, *Animal Ecology*, in which Elton described the role that competition for food and other economic resources within ecological niches played in the regulation of animal numbers, and his *The Ecology of Animals*, in which, focusing on overcrowding and “over-parasitization,” he outlined a theory in which epidemics acted as “automatic checks” on animal populations (Anderson 2016, pp. 247–248; Elton 1927, 1966, pp. 54–55).^18^ The key point is that the frame through which Burnet viewed these ecological interactions was at the level of populations, not the individual. In this way, Anderson argues, his ecological studies helped underwrite “a complex, biologically informed epidemiology” (Anderson 2004, p. 39).

However, Burnet’s epidemiological focus also meant that he tended to see ideas of immune balance in similar population-wide terms. This conception did not extend to the level of the ecology of the individual, however. Here, as Swiatczak has shown, ideas of balance ran counter to the other strands of Burnet’s ecological and immunological thought, notably his clonal selection theory of antibody production with its paradigmatic mechanism of self/nonself discrimination (Swiatczak 2014). As we shall see, this set Burnet on a very different ecological trajectory to Dubos.

Zabusky speculates that Dubos may have originally learned of Burnet’s work through a lecture at the Harvey Society in 1940 in which Meyer, who had begun corresponding with Burnet in the 1930s, had referred to Burnet’s studies of psittacosis and suggested that infection might be more profitably studied “as a branch of academic biology” (Zabusky 1986, p. 70; Meyer 1939–1940, p. 92).^19^ Before recommending Burnet as a Dunham Lecturer, Dubos must certainly have familiarised himself with Burnet’s work on virus cultivation and virus diseases as his correspondence with the chair of the selection makes it clear that he held Burnet in the highest regard (*op. cit*., note 15). Certainly Burnet’s lectures, which were attended by more than 400 people, reflected well on the medical faculty. Afterwards, Dubos hosted a lunch party for Burnet and they continued their discussions, prompting Burnet to describe Dubos as a “kindred soul.”^20^

It was also at Harvard that Dubos met the biologist and chemist Jeffries Wyman and was challenged by Wyman to explain the basis of Pasteur’s influence and fame “since he never did discover any fundamental laws for which the nineteenth century is so well known” (Benison 1957 II, p. 2). According to Moberg, as a student Dubos had been sceptical of claims that Pasteur should be viewed as a scientific visionary and great benefactor to mankind (Moberg 2005, pp. 98–99). Writing Pasteur’s biography in 1948 gave Dubos a new appreciation of the French microbiologist and the tension he detected between Pasteur’s experimental choices and the wider scientific questions that informed his pursuit of knowledge. However, it was not until 1976, when Dubos came to write a new introduction to the book and had a chance to reflect on the parallels with his own career, that he would also realise that Pasteur’s science had contained ideas similar to his own and argue that he deserved to be seen as an ecologist manqué (Dubos 1976a).

Trained in physics and organic chemistry, Pasteur had been studying crystals and optical rotation when he became fascinated by fermentation and suggested a spontaneous role for microbes. From this he was drawn inexorably to the study of the microbial causes of infectious disease and the development of vaccines against anthrax and rabies, but, Dubos argued, at almost any point in his scientific career Pasteur could have taken a different path. From the beginning of his biological investigations, Pasteur had realised that the morphology and chemical activities of microbes were profoundly influenced by their environment, Dubos explained. Indeed, in his very first paper on the fermentation of lactic aid, Pasteur had observed how bacteria responded differently depending on whether solutions were acid or alkaline. Similarly, in his studies of silkworms he found that the same microbe could cause two different diseases, *pébrine* and *flacherie*, depending on the levels of heat, humidity, oxygen and nutrition. This preoccupation with the physiochemical characteristics of microbial environments, or what Pasteur referred to as the “terrain,” never left him. “I now see more clearly than I did when writing Pasteur’s biography that the magnitude of his theoretical and practical achievements derives in large part from the fact that his conceptual view of life was fundamentally ecological,” Dubos asserted in 1976 (Dubos 1976b, p. xxx).

## Tuberculosis and the “extremely complex property” of virulence

Whatever the influence of Pasteur, Burnet, and the other thinkers he encountered at Harvard, by the time Dubos returned to the Rockefeller Institute in July 1944 microbes were no longer his central concern. Instead, the study of microbes had become a means to answering wider questions about the nature of virulence and the influence of physiological and other host conditions on resistance and susceptibility to disease. Dubos would refine these thoughts in *The Bacterial Cell*, which was published after his return to the Rockefeller, but his earliest statement of his new approach came at a symposium on wartime advances in medicine in New York in April 1944, 3 months before he left Harvard, in which he surveyed some of the research “trends” he considered important to the future control of infectious diseases (Dubos 1944). Foremost amongst these was a better understanding of “the nature of virulence and the genesis of epidemics” (p. 208). The achievement of vaccines against smallpox, yellow fever, plague, and tuberculosis, Dubos believed, coupled with Pasteur’s brilliant showmanship, had blinded medical researchers to the limitations of germ theory. The discovery of the etiological agent of an infection was only the first step to understanding the course of infectious disease processes. In future, he predicted, the development of reliable vaccines and long-lasting chemotherapeutic agents would depend on a better understanding of the “many sided relationship between the pathogen and the host” and the “extremely complex property” of virulence (p. 208). For instance, in vitro studies had shown that the virulence of streptococci was associated with certain cellular constituents of the bacteria. However, the possession of these cellular elements was not sufficient to make the bacteria “fully virulent,” but was dependent on various “elusive elements” that were lost when the bacteria were cultivated in artificial media (p. 209). Moreover, highly pathogenic strains might possess a low degree of communicability, and, conversely, highly communicable strains might cause little disease. As an example, Dubos pointed to the fact that, during the war, it had been noted that strains of streptococci and meningitis were frequently present in military camps without giving rise to outbreaks. It was also well known that some families exhibited greater susceptibility to tuberculosis. Moreover, there was reason to believe that nutritional deficiencies, hormonal imbalances, and stresses associated with war also played a role in resistance and susceptibility to infection (Dubos was no doubt thinking of the death of his wife here). Instead, Dubos urged a closer study of the physiological factors governing host resistance, suggesting that it was “through a disturbance of the normal physiological processes of the host that pathogenic organisms cause those symptomatic and pathological manifestations which characterize each individual infectious disease.” Ignorance of these factors, he thought, was due to the fact “since the beginning of the microbiological era, the study of infection has been almost exclusively limited to the immunological aspects of the host–parasite relationship.” In short, it was only by “returning to the main channels of biological and biochemical philosophy [that] the student of infection will achieve a more complete picture of the many reactions by which the host responds to the special stimuli exerted by the parasite.” (pp. 212–213).

In theory, Dubos’s interest in bacterial variation and virulence put him closer to the British tradition of experimental epidemiology, with its emphasis on the incorporation of statistical methods into bacteriology, than the thought styles and experimental methods that predominated at the Rockefeller. Whereas British interwar epidemiologists had sought to explain the rise and fall of epidemics in terms of disturbance of equilibrium models and by embracing the possibility of variations in bacterial virulence, Rockefeller researchers had stressed the homogeneity of bacteria and argued that factors other than changes in virulence were responsible for epidemics. As Amsterdamska has argued, this had as much to do with differences of “thought style” and the Rockefeller’s preference for laboratory studies where variations in host susceptibility could be keep to a minimum by using standardized mouse populations bred expressly for experimentation. By contrast, British researchers thought that the Rockefeller’s procedures for testing virulence and culturing bacteria were too removed from the conditions that pertained in nature and might not give an accurate picture of human epidemics (Amsterdamska 2004, pp. 499–503). Ironically, Dubos who shared this scepticism about the laboratory as an analogue for nature, would use similar bacteriological methods and laboratory-bred mouse populations to explore the very questions that had animated British experimental epidemiologists: namely, the relationship of bacterial virulence to changes in host resistance or susceptibility and changes in the environment.

Encouraged by the institute’s new director, Herbert Gasser, a biologically minded physiologist and Nobel laureate, Dubos decided to focus on the metabolism of the tubercle bacillus with a view to better understanding the pathogenesis of the disease. To staff his laboratory he brought two researchers who had gone with him to Boston, Cynthia Pierce and Letha Jane Porter (Porter had read *The Bacterial Cell* in draft and would become Dubos’s second wife in 1946). Other key recruits included Gardener Middlebrook, a physician from Harvard; Merrill Chase, a researcher recruited from Karl Lansteiner’s laboratory; and Bernard Davis, a physician and bacterial geneticist on loan from the United States Public Health Services’ new Tuberculosis Control Division.

Dubos initially embarked on a comparative study of the physiology of virulent and avirulent tubercle bacteria with a view to discovering a specific component that rendered the bacillus dangerous. The study was explicitly modelled on Avery’s investigations of the polysaccharide capsule of the pneumococcus. In 1917, Avery and Alphonse Dochez had demonstrated that pneumococcal bacteria were type specific and that this specificity was due to a soluble substance—SIII for short—that was an essential component of virulence. Avery and his co-workers further demonstrated that this substance was composed of polysaccharides that enabled the pneumococcus to resist phagocytosis and which gave the capsule its characteristic smooth and shiny appearance.^21^ At the time, the finding that not only proteins but carbohydrates were involved in virulence was a major advance. But as Dubos explained in his 1948 lecture, even more important was the way that Avery’s immunochemical approach had shown that virulence and immunity could be analyzed in terms of a “few highly specialized components” of parasitic cells (Dubos 1948, p. 176). Dubos hoped that, as with his discovery of the SIII enzyme, this approach would yield a reliable prophylactic against tuberculosis. One problem with the BCG vaccine was that it had been produced from a live attenuated strain of bovine tuberculosis, presenting a theoretical risk that it could return to virulence, and had never gained wide acceptance in the US due to opposition from the Trudeau Society (*op. cit*., note 1). If Dubos could identify a chemical constituent of the human tuberculosis bacillus that protected it from attack, he theorized it might be possible to induce a state of immunity using killed tubercle bacteria, which would be safer than a live vaccine. At the same time, spurred by Marie Louise’s death, Dubos set out to discover what disturbances in the host’s environment converted a latent infection into an active case of tuberculosis.

In addition, Dubos decided to investigate a more mundane aspect of tuberculosis research: namely, the difficulty of culturing tubercle bacilli in the laboratory. *Mycobacterium tuberculosis* is a rod-shaped bacillus notable for its thick waxy cell wall and slow growth. Under optimal laboratory conditions it requires up to 24 h to undergo one cycle of replication and it can take 2 weeks for a visible colony of mycobacteria to appear on a solid culture medium. The bacillus is also aerobic—it reproduces best in tissues such as the lung that are rich in oxygen, and also spreads easily in the air. In Dubos’s laboratory, where experiments were conducted at open benches and researchers often used unplugged pipettes, this made it an extremely risky organism to work with.

In order to conduct quantitative experiments, Dubos required a uniform colony of bacteria, but on standard culture media the bacilli grew slowly in large undifferentiated clumps, meaning mycobacteria inside the clumps were exposed to different growth conditions than those on the outside. While visiting the laboratory at the sanatorium in the Adirondacks where Marie Louise had convalesced in 1942, Dubos had observed that this clumping was due to fatty substances on the surface of the bacilli that resisted wetting. In order to study the bacilli, microbiologists ground these clumps, or pellicles, into smaller and smaller particles. However, Dubos observed, this operation was “rather clumsy and somewhat dangerous” and led to the mixing together of bacteria that had grown under “extremely different environmental conditions” (Benison 1957, II, p. 11). The result was a heterogeneity of cultured strains, whereas what was needed was a way of fostering the diffuse growth of homogenous bacilli, i.e. either entirely virulent or avirulent bacteria.

In 1946 Dubos solved this problem by adding a commercial detergent, Tween, to the growth media, enabling the production of uniform cultures of young mycobacteria that he separated into virulent and avirulent groups based on observable morphological and chemical differences (virulent bacilli grew in serpentine cords and bound to neutral red dyes, whereas avirulent bacilli grew without any orientation) (Dubos and Davis 1946; Dubos and Middlebrook 1948; Middlebrook et al. 1947). This was a major technical advance in that it gave highly reproducible results and in succeeding years the new growth media were used to develop more rapid assays for tuberculosis, to test the mycobacteria for drug sensitivities, to explore the viability of strains of the BCG vaccine, and to search for virulent and immunogenic components. By the later 1940s they were also being used to establish experimental infection models in mice. In these endeavours Dubos was greatly aided by another recruit to his laboratory: Frank Fenner.

In 1948, Burnet had written to Dubos to ask whether he would be willing to host Fenner at his laboratory. By that time Fenner had been at the Walter and Eliza Hall Institute in Melbourne for 2 years. There, he had impressed Burnet with his studies of ectromelia, a virus that Burnet had established was related to smallpox and which Fenner had demonstrated produced a similar pox-like rash in mice, and Burnet was now eager for his protégé—“the best recruit we have had to microbiology in Australia for ages”—to gain experience abroad.^22^ Dubos was happy to oblige and in the spring of 1948 Fenner arrived at the Rockefeller on an 11-month fellowship. He found his new working conditions liberating. “Dubos was quite different from Burnet,” he wrote. Whereas Burnet was a “dominating” hands-on scientist, Dubos “didn’t work at the bench at all, himself.” Instead, Dubos surrounded himself with a small group of post-doctoral students. “At the end of the day he would ask everybody what they were doing and erect an inverted pyramid of speculation on a point of fact,” Fenner recalled. “It would often collapse, as you can imagine” (Blyth 1992–1993). Nonetheless, Fenner found Dubos “a man of wide vision,” one who, “like Burnet… combined an interest in the specific properties of microorganisms with a deep appreciation of the ecology of disease.” Both men were also “exceptional talents, each of whom saw much further than the immediate laboratory experimentation of which they were nevertheless masters”^23^ Little wonder then that Fenner would later describe Burnet and Dubos as his most important intellectual influences and keep pictures of both men on his desk at the John Curtin School of Medical Research, in Canberra.^24^

Knowing that Dubos was interested in mycobacteria, Fenner brought two strains recently isolated in Melbourne, *M. ulcerans* and *M. balnei*. These mycobacteria proved “very interesting” in that they produced severe skin lesions but due to temperature restrictions did not multiply when taken into the body (Blyth 1992–1993). Soon Fenner was making other contributions to Dubos’s laboratory. Refining Dubos’s new rapid dispersed culture method for tuberculosis, Fenner first helped develop a more reliable assay for counting viable tubercle bacilli (Fenner et al. 1949). Next, he and Dubos adapted the dispersed culture technique to measure the biological effects of the bacilli in the BCG vaccine. In so doing, they demonstrated for the first time that the vaccine contained several substrains that differed in their morphology, growth requirements and immunizing power. Not only that but the vaccine was unstable, containing mixed populations of virulent and avirulent bacilli. Subsequent experiments conducted by Dubos’s laboratory suggested that BCG achieved its immunizing effect by encouraging the BCG culture to multiply in host tissues where it caused a self-limiting disease. This meant that the most virulent bacilli were both the most effective and, potentially, the most dangerous (Dubos et al. 1950b). After making this discovery, Dubos, Fenner, and Pierce developed guidelines for the standardization of BCG vaccines worldwide (Dubos and Fenner 1950; Dubos et al. 1950a). Since then it has been found that while the vaccine does not prevent the reactivation of latent pulmonary TB, it protects children against disseminated TB in the environment and is extremely safe, hence its continued recommendation by the WHO in national childhood immunization programmes (Tang et al. 2016).^25^

Dubos’s group also demonstrated that virulent bacilli were immediately immobilized by the host’s leucocytes, while avirulent bacteria were unaffected, and developed a test—known as the “cord factor test”—which showed that virulent bacilli grew in long serpentine cords, while avirulent strains grew in a disoriented manner in clumps (Middlebrook et al. 1947; Martin et al. 1950). Dubos further demonstrated that certain substances present in the in vivo environment, such as short chain organic acids, exerted an inhibitory effect on the growth of tubercle bacilli, while other substances, such as serum albumin, promoted their growth. In this way, Dubos was able to show that the “physiochemical environment prevailing in and around the tuberculosis lesion is of paramount importance in determining the course of the infectious disease process” (Dubos 1952, p. 640).^26^

However, perhaps Dubos’s most significant contribution came from his exploration of the physiological characteristics that govern a host’s susceptibility and resistance to infection. The animals chosen for these experiments were a colony of albino mice, labelled NCS, that had been maintained continuously at the Rockefeller for 40 years and which Dubos had selectively bred in germ-free conditions.^27^ Using these colonies, Dubos was able to show that certain strains of tubercle bacilli were virulent in the NCS mice while others were not. By controlling their intake of nutrients, he also demonstrated that the composition of diets markedly affected the survival of infected mice by causing non-specific physiological disturbances or stresses (Dubos and Pierce 1948). Other non-specific stresses included inoculation with bacterial endotoxin (Schaedler and Dubos 1961). Such stresses did not involve localized mechanisms but instead created an “in vivo environment favorable for the survival and proliferation of staphylococci and tubercle bacilli, as well probably as of other microbial agents” (Smith and Dubos 1956, p. 118). Moreover, whereas mice placed on restrictive diets were initially susceptible to disease, they rapidly recovered their resistance once they had adapted to their new dietary environment (Schaedler and Dubos 1956).

This research was crucial to Dubos’s developing ecological ideas as it pointed to the role of wider environmental factors in the disturbance of biochemical processes involved in the regulation of equilibrium states. In *The White Plague*, the Duboses had highlighted the role of social, economic, cultural and psychological factors in the manifestation of tuberculosis through the ages (Dubos and Dubos 1987). The central message of the book was that tuberculosis could not be explained by the presence of the microbe alone and that it was only through long familiarity with the disease that communities had evolved resistance. As Dubos told an interviewer in 1957: “These tubercle bacilli are … all around us, they are a ubiquitous component of our environment. The very fact that man has survived as a species means that through ordinary adaptation he has achieved some sort of balance, or equilibrium with the tubercle bacillus (Benison 1957 V, p. 151).

Dubos was well aware that his germ-free mice could not be taken as an analogue for nature and that tuberculosis might behave very differently under natural conditions and in other animals. As he informed the same interviewer, the mice “merely serve as experimental models to study the agencies or the accidents of human life that disturb the normal state under which we can be at peace with the potentially pathogenic agents in our environment.” By the later 1950s, however, Dubos could also cite evidence for his views from an “amazing” natural experiment underway in Australia conducted by his former laboratory assistant (Benison 1957 V, p. 154).

On returning to Australia in 1949, Fenner had become professor of microbiology at the John Curtin School, in Canberra. There, at Burnet’s instigation, in 1951 he launched a ground-breaking study of myxomatosis in Australia’s wild rabbit population, showing how under conditions of epizootic transmission attenuated strains of the myxoma virus enjoyed a selective advantage, most likely because by enabling their hosts to live longer they afforded greater opportunities for onward transmission of the virus by mosquitoes (by contrast, virulent strains, by killing rabbits rapidly, afforded fewer opportunities for horizontal transfer). Dubos was greatly interested in Fenner’s study of what he called “host–parasite ecology.”^28^ When myxomatosis was first introduced to Australia in 1950, the virus had proven highly lethal, but by the time Fenner became involved there were suggestions that it was becoming less so, though whether this was due to a variation in the virulence of the virus or genetic factors was unclear (Anderson 2017). Dubos quickly grasped the significance of Fenner’s study. “For all the reasons that you know, I am most excited in your story of the outbreak of myxomatosis,” he wrote Fenner in March 1951. “I hope you will keep me informed.”^29^ Following his return to Australia in 1949, Fenner had continued exchanging notes on tuberculosis with Dubos, with Dubos informing Fenner in May 1950 that he was close to demonstrating how inflammatory reactions interfered with the growth of virulent strains of the bacilli.^30^ In response, Fenner began sketching a review of the bacteriological and immunological aspects of BCG vaccination and went on to praise Dubos’s mice studies as approaching “closer to the ideal method of determining virulence”.^31^ Fenner completed the review for the journal *Advances in Tuberculosis Research* just in time as by March 1951 all his energies were need for his myxomatosis study (Fenner 1951; Fenner & Ratcliffe 1965). What made Fenner’s study of myxomatosis so important to Dubos was that it provided a model for how other formerly virulent microbial diseases, including tuberculosis, had first presented in human populations before evolving towards equilibrium. Thus, addressing the Canadian Medical Association in 1958, Dubos described how when plains Indians migrated to a reservation in Saskatchewan in the late nineteenth century the annual death rate from tuberculosis had been 90% and more than half the families had been eliminated in the first three generations. However, by the fourth generation mortality from tuberculosis had fallen substantially and only 1% of Indian children raised on the reservation were exhibiting signs of illness. Eskimos and Polynesian islanders had suffered similarly dramatic die-offs when first confronted with pathogens introduced by European and American settlers. The only reason why tuberculosis presented a less acute problem in the Western World than in the past, Dubos explained, is “that we are fortunate beneficiaries of the tremendous selective process brought about by the widespread epidemics of a few generations ago” (Dubos 1958, p. 448). However, as Dubos warned in a popular article in *Scientific American*, such infections persisted in a latent state and resistance to them could only be counted on in the “narrow range of conditions constituting the ‘normal’ environment in which the population has evolved” (Dubos 1955, p. 34). “Any shift from the normal is likely to render the equilibrium unstable,” he argued, before concluding that virulence could no longer be regarded solely as a property of microbes but was now “coming to be thought of as ecological” (pp. 34–35).

## The ecological aspects of host–parasite relationships

From the above, it would be tempting to conclude that by the middle 1950s Dubos had come over to Burnet and Fenner’s views of disease ecology. However, this would be to misconstrue the sense in which Dubos employed ecological terminology and sought to distinguish his ideas from those of his contemporaries, as well as his intellectual antecedents. The clue comes from the passage from Dubos’s 1948 Avery lecture quoted earlier in this paper in which he lamented that microbiologists “know much of the *ecological aspects of host*–*parasite relationships* in tuberculosis, hardly anything *of the means used by the bacillus to behave as a parasite*” [italics inserted] (Dubos 1948, p. 179). Why the juxtaposition if Dubos considers his views synonymous with an ecological approach to parasitical microbial infections? The answer, I believe, is that while Dubos accepted that virulence could not be understood apart from macro environmental forces and processes of natural selection, throughout his career he tended to privilege biochemical explanations for commensal parasitic states of infection without disease. Crucially, this not only put him at odds with Burnet, it also put him at variance with Smith. Indeed, Dubos began the O. T. Avery lecture by praising Smith’s insights into equilibrium states, only to write in the very next sentence that although “this broad biological and ecological point of view has been extremely useful in the analysis of epidemiological problems…it has contributed little to the understanding of the mechanistic aspects of parasitism.” It was in this context that Dubos thought microbiologists needed to know more about the “means used by the bacillus to behave as a parasite.”

For a more a detailed account of Dubos’s approach and to understand the sense in which he was happy to be called a disease ecologist we must turn to *Biochemical Determinants of Microbial Diseases* (Dubos 1954). Based on material that Dubos had presented at the Warren Triennial Lectures in Boston in 1953, and refined during his visiting professorship at the University of California, Berkeley, in the spring of 1954, this was Dubos’s first scientific book since *The Bacterial Cell* and the clearest signal yet that he now considered himself a medical researcher. At Berkeley, Dubos had become reacquainted with Meyer’s research on psittacosis and his survey of latent infections (Meyer 1936). Indeed, Dubos cites Meyer’s survey early in the first chapter, “Infection into Disease.” He also draws on Smith’s writings on parasitism and for the first time cites Burnet’s *Virus as Organism* (Burnet 1945). However, it is to Bernard that Dubos turns to for an antecedent for his ideas about the importance of the physiochemical environment of the host to the pathogenicity of microbes, and to Winogradsky for an ecological methodology that will shed light on these interactions. Usually, Dubos explains, parasites exist in a state of latent infection. “Only when something happens which upsets the equilibrium between host and parasite does infection evolve into disease. In other words, infection is in many cases the normal state; it is only disease which is abnormal” (Dubos 1954, p. 2). Traditionally, microbiologists had offered an explanation of latent infections based on “immunological techniques” (p. 3). However, Dubos argued, it was not enough to analyze the “host–parasite relationship” in terms of antibodies and acquired immunity; just as important was the in vivo biochemical environment.

As one tries to discover a metabolic basis for pathogenicity, it soon becomes apparent that the first question to be answered is not why pathogens can cause disease, but rather why saprophytes do not proliferate as well—or at all—*in vivo*… The answer to this riddle will certainly be found in one aspect of the problem which is rarely mentioned and never studied, namely, the very special types of environment which microorganisms find in animal tissues (p. 13).

Dubos argued that microbiologists needed to pay attention to two kinds of in vivo environments. One was “the extracellular environment in which blood and tissues are bathed under normal conditions”—in other words, the blood and lymph systems captured in Bernard’s concept of the *milieu interieur* (p. 14). The other was the intracellular environment “before phagocytosis, inflammation, and necrosis have occurred” (p. 22). This was likely just as important to the outcome of infectious disease processes, but due to a paucity of knowledge of these intracellular environments and the mechanisms used by parasitic bacilli it was difficult to know what role they played in pathogenicity. Just as Winogradsky had shown that soil microbes could only be understood in their natural environment, so, Dubos argued, the study of infectious disease must be placed on a similar “ecological basis”:

No metabolic analysis of infectious disease is possible until an ecological concept is introduced to formulate the problem. It is because this ecological concept has been lacking almost completely heretofore that bacterial biochemistry has contributed so little to the understanding of pathogenesis (pp. 22–23).

The significance of this passage cannot be overstated as it points to the essential contrast Dubos sees between his approach and those of other medical microbiologists in the period who, for all their insights into the biology and ecology of host–parasite interactions, have yet to apply adequate ecological methods to the study of bacterial biochemistry. It is in this narrow methodological sense that Dubos is happy to describe himself as an ecologist. In one sense, this passage can be seen as a reassertion by Dubos of the importance of in vivo studies, the difference being that he now has a decade of tuberculosis research to draw on in addition to his work on bacterial enzymes. However, Dubos is also signalling an important departure in his thinking along new ecological lines. The clue comes in the next paragraph where, citing the work of the plant pathologist R. W. Lewis on the nutritional requirements of parasites, Dubos argues that equilibrium states depend on “the proper balance between different biochemical factors” (p. 24). In his paper, Lewis had surveyed a series of studies showing how variations in the nutritional environment of plants altered the composition of important metabolites and hence the plants’ susceptibility to a range of bacterial and fungal infections. Lewis argued this supported the theory that under normal conditions there was a balance between host and parasite which reflected their long evolutionary history (Lewis 1953). Lewis noted that Dubos’s studies had similarly pointed to the role of diet and nutrition in inhibiting or encouraging the growth of tubercle bacilli and other quiescent bacterial infections. Now, in an analogous way to plants, Dubos suggested that in order to understand biochemical changes that might alter the equilibrium between a host and parasite at a micro level it was also necessary to study the wider macro forces or “non-specific stress” operating on an organism. These stresses could include changes in temperature, hormonal disturbances, and emotional or “nutritional upsets,” and applied particularly to the revival of chronic infections, such as herpes simplex, or infections due to intracellular parasites, such as psittacosis and rickettsia (Dubos 1954, pp. 1, 4, 114). Dubos suggested that ideas of specific acquired immunity of cellular immunity were insufficient to explain resistance to infection and that inflammatory responses might be equally important to the regulation of equilibrium states, being “a manifestation of the wisdom of the body designed to maintain the status quo against the noxious influences of the outside world” (p. 120). In 1956, in a reworking of the same themes in a paper presented at a conference on psittacosis, Dubos was even bolder. Beginning with a survey of conditions in which various forms of physiological stress appeared to play an important role in the manifestation of disease, Dubos cited the eruption of herpetic blisters due to fever, tuberculosis brought on by “gross malnutrition,” and the activation of latent psittacosis infections in birds due to the stresses associated with breeding in overcrowded aviaries.^32^ Similarly, there was evidence that the overuse of antibiotics could make individuals more susceptible to infection with commensal fungi and bacteria, while similar pathological processes had been observed in the intestinal flora of laboratory mice exposed to “excessive radiation” (pp. 5–6). Such observations pointed to the fact that immunological factors were insufficient to account for latent infections. Such balanced states “might be expected to persist only as long as the *milieu interieur* of the host remains constant or at least within normal limits,” Dubos explained. However, this supposed constancy could not be taken for granted.

By conditioning the composition of the internal microenvironment of the host, the external macroenvironment can … upset the operations of the evolutionary forces that normally restrain the microbial agents *in vivo*. Thus, it is not surprising that the disturbances caused in the microenvironment by the macroenvironment often determine whether infection remains silent, or expresses itself in overt disease.

In short, it was time for medical researchers to embrace a “broader understanding” of infectious disease, “one in which the Pasteur–Koch philosophy would be supplemented by that associated with the names of Darwin and Claude Bernard” (pp. 16–18). This insight would increasingly inform Dubos’s research at the Rockefeller from 1960 onwards, a period which saw him change the name of his laboratory from Bacteriology and Pathology to Environmental Biomedicine and in which he embarked on a study of the microbial flora of the gastrointestinal tract and role of microbiota in the regulation of healthy physiological function, what would come to be seen as a forerunner of interest in the human microbiome.^33^

## Conclusion

Dubos’s decision to abandon antibiotics research in the 1940s and study the tubercle bacillus puzzled his contemporaries, but we can now see how his fascination with tuberculosis was prompted not only by his wife’s death but was a logical extension of his research into soil enzymes. These studies began with pneumococcal disease and the hunt for an enzyme that would make the pneumococcus susceptible to attack, but by the 1940s had led him to look into the chemical constituents of the tubercle bacillus and the physiochemical determinants of virulence.

Dissatisfied with the germ theory model which tended to see virulence as a property of the microbe and its “power to invade” animal tissue, Dubos initially saw virulence as the product of chemical constituents of bacteria and complex biological interactions between hosts and parasites occasioned by changes in the micro-environment in which microbes resided within animal hosts. However, by the later 1940s, influenced by his inquiries into the social history of tuberculosis and his exposure to Burnet’s scientific ideas, Dubos was increasingly looking to wider macro-environmental and evolutionary forces to explain the virulence or avirulence of microbes and manifestations of disease. Using germ-free laboratory mice, Dubos devised experiments to examine the role of nutrition and non-specific environmental stresses in resistance to disease and the reactivation of latent tuberculosis infections. At the same time, he entered into a correspondence with Burnet’s protégé Fenner, whose study of myxomatosis Dubos saw as an analogue for the variations in virulence he had observed in the laboratory and corroboration of evolutionary processes and equilibrium states in nature. In this way, he was gradually able to tease out the “complex property” of virulence and incorporate evolutionary and scientific ecological perspectives into his thought.

Dubos’s later research and writings are beyond the scope of this paper. Suffice to say that one can trace a direct line from his laboratory research in the 1940s and 1950s to his mature ecological philosophy. Dubos’s first expression of his new vision came in his 1959 book *Mirage of Health*, in which, drawing on his research into tuberculosis and antibacterial resistance, he argued that attempts to eradicate infectious disease were doomed to failure “because at some unpredictable time and in some unforeseeable manner nature will strike back.” Instead, he emphasized the harmony of the organism with its environment and preached peaceful co-existence with microbes, arguing that humans were part of a “complex ecological system” (Dubos 1996, pp. 266–267). However, by the time he came to write *Man Adapting* in 1965, spurred by his research into indigenous microflora, he was increasingly coming to be see disease as a failure to adapt to environmental insults of man’s own making and his ecological analysis was being supplemented by humanistic value judgements (Dubos 1965). It was a theme that Dubos would continue to explore for the rest of his life in popular books such as *So Human an Animal* and *Man, Medicine and the Environment* (Dubos 1968a, b). The result was a rejection of ideas of struggle and economic competition that were becoming increasingly central to scientific ecology. Instead, Dubos came to emphasize the “symbiosis between humankind and earth” (Dubos 1976a).

This paper has shown how the trajectory of Dubos’s ecological thought was dictated both by his working practices as an experimental biologist at the Rockefeller and his own ecology of knowledge, to invoke Rosenberg’s useful phrase (Rosenberg 1995). In recent decades scholars have traced these ecologies of knowledge to, variously, bacteriological epidemiology (Mendelsohn 1998) parasitology (Farley 1992) and tropical medicine (Worboys 1998; Tilley 2011). In addition, historians have shown how ecological conceptions of infectious disease were influenced by medical researchers’ encounters with novel pathogens endemic in “settler societies” (Griffiths and Robin 1997; Mitman 2005; Nash 2006). This was particularly true of Burnet, whose work on behalf of the Council of Scientific and Industrial Research in Australia fostered an interest in the control of agricultural pests and animal viruses, prompting interdisciplinary collaborations with parasitologists, zoologists and animal ecologists. And it was also true of veterinary pathologists, such as Smith and Meyer, whose work on behalf of state and agricultural bureaus in the United States brought them face to face with diseases of livestock and pathogens that posed a threat to the livelihoods of farmers and cattle ranchers.

By contrast, despite being educated at a French agricultural college, Dubos had little experience of or interest in veterinary medicine. Nor did he have the opportunity, as Fenner and Meyer did, to study the interaction of animal viruses and their vectors in the field in complex ecological settings. Instead, Dubos worked almost exclusively on bacterial diseases that were directly transmissible to humans and which could be modelled in the laboratory. This set Dubos on a very different track to Fenner and Burnet whose interest in host–parasite interactions under natural conditions led them to look to animal ecology for a theoretical framework with which to make sense of their epidemiological and immunological observations. Instead, Dubos turned for inspiration to Winogradsky, Bernard, and Pasteur, and to his social and historical investigations of the role of environmental factors in the regulation of health and disease.

At the same time, inspired by Avery’s immunochemical approach to virulence, Dubos became increasingly interested in the biochemical or mechanistic aspects of parasitism. In contrast to Burnet and Fenner’s epidemiologically oriented ecology, this led Dubos to focus on the interface of hosts and parasites at the micro level of individuals. Crucially, these local environments operated *within* phagocytes and hence Dubos considered that they fell within a different ecological realm to population ecology. However, to the extent that local ecological interactions were conditioned by the entire physiological metabolic state of an individual they were also subject to wider ecological and immunological conditions. Indeed, Dubos considered that these local and whole body systems were linked, such that external stresses operating on the individual could upset normal physiological regulation in such a way as to throw these local ecologies out of synch. This is what Dubos meant when, invoking the other sense of ecology, he stated that “any metabolic analysis of the infectious process must be placed on an ecological basis” (Dubos 1954, p. 22).

It would be interesting to go on to explore the subsequent development of Dubos’s ecological thought in the 1960s. Already, by 1958, in a paper reworking the ideas outlined in the first chapter of *Biochemical Determinants*, there is evidence that he was coming to eschew ecological terminology in favor of an emphasis on balance and the importance of adaptive mechanisms to restoring equilibriums disrupted by “endogenous or exogenous” insults (Dubos 1958, p. 433). Certainly, Dubos’s realization that organisms have multiple potentialities and are continually making creative adaptations would increasingly inform his later ideas about human health and what Moberg calls his “wholistic biology” [sic] (Moberg 2005, p. 133). The result was that while Dubos never fully engaged with the science of ecology, he was able to incorporate ecological ideas into his thought and practices, and relate them to his views about the natural harmony of man and his environment. However, while Dubos was happy to employ ecological terminology from time to time and associate himself with ecological currents in epidemiological thought, he remained deeply ambivalent about ecological language. Instead, for Dubos ecology served primarily as metaphor, one that enabled him to connect his scientific work to his broader humanistic concerns. As he put it a 1976 paper for the Society of American Bacteriologists comparing his career and research choices to those of his hero Pasteur, “By choice, I have increasingly devoted my professional life to the study of the interplay between organisms and environment—call it ecology if you will” (Dubos 1976b, p. 703).

## Footnotes

1 R. Dubos, “The mortality of tuberculosis.” Lecture delivered before the New York branch of the *Society of American Bacteriologists*, 5 January 1950, p. 2. René Jules Dubos Papers, Rockefeller Archive Center. RU 450 D851, Box 25, Folder 3.

2 The vaccine was named for the French scientists, Albert Calmette and Camille Guérin, who isolated it from an attenuated live *Mycobacterium bovis* strain in 1919. Although by the 1921 the strain had showed no reversion to virulence after 230 passages on a bile-potato medium, in 1948 Calmette and Guerin recognised that protection was “relative” and that a subject challenged with massive, repeated and/or highly virulent tubercle bacilli could develop tuberculosis reinfection (Gheorghui 1995).

3 Dubos, “The mortality of tuberculosis,” p. 4.

4 R. Dubos, “The effects of events, surroundings and personal choices on the scientific interests and attitudes of René Dubos at age 81.” Unpublished manuscript, 1981, p. 17. René Dubos Papers, Rockefeller University Archives. RU450 D851, Box 31, Folder 14.

5 In a note appended to a manuscript in 1959, under the heading “further reading,” Dubos described the 1953 edition of *Biological Aspects*, which now carried the revised title, *Natural History of Infectious Disease*, as “rather technical, but nevertheless highly readable and exciting” R. Dubos, “The Scientists Speak: Biology,” unpublished manuscript for Harcourt, Brace and Co., 1959. René Dubos Papers, Rockefeller University Archives. RU 450 DA51, Box 26, folder 22.

6 F. M. Burnet, “Some virus diseases of Man—evolutionary and ecological considerations,” Edward K. Dunham Lectures for the Promotion of the Medical Sciences, 1943–1944. Archives, Countway Library, Harvard Medical School.

7 R. Dubos, “Post prandial musings on resistance to infectious diseases.” Lecture to Reed College Symposium, Frederick, Maryland, September 1959. René Dubos Papers, Rockefeller University Archives. RU 450 DA51, Box 26, Folder 25.

8 For a summary of Bernard’s theory of the *milieu interieur* and its continuing relevance to medicine and the life sciences see Conti (2001) and Robin (1979). Winogradsky developed his ecological ideas in a series of papers published prior to and during World War II (Winogradsky 1924, 1938, 1942).

9 “Pneumonia yields to new chemical,” *New York Times*, 9 September 1939, p. 19.

10 Dubos, “The effects of events, surroundings and personal choices on the scientific interests and attitudes of René Dubos at age 81,” p. 22.

11 It was only with the establishment of the Rockefeller University Archives in 1974 that Dubos was persuaded to save important correspondence and manuscripts.

12 Dubos was referring to physiological adaptations, rather than evolutionary adaptations in the Darwinian sense.

13 According to Joshua Lederberg, the Nobel Prize-winning bacterial geneticist and Dubos’s former student, *The Bacterial Cell* influenced a generation of medical researchers and brought into “sharp focus what we now take for granted—namely, that bacteria are cells.” (Lederberg 1990).

14 Dubos, “Post prandial musings on resistance to infectious diseases,” pp. 2–4.

15 In a letter to A. Baird Hastings, the head of the Dunham selection panel, Dubos wrote: “I know of no candidate who would be more suitable as a Dunham lecturer.” Six days later, another panel member, Joseph C. Aub, reported: “Dr Dubos is enthusiastic about Burnet’s work. He does not know him personally but work should be the criterion of a Dunham lecturer, not personality… I have not seen [Burnet’s] bibliography, but I would take Dr. Dubos’s opinion.” Dubos to Hastings, 14 September 1943; Aub to Hastings 20 September 1943. Frank Macfarlane Burnet papers, Baillieu Library, University of Melbourne, “Correspondence re: Dunham Lectureship.”.

16 Frank Macfarlane Burnet, “Notes on American Visit”. Typed diary notes—American tour, 4 December 1943–1944. Frank Macfarlane Burnet papers, Baillieu Library, University of Melbourne.

17 The example Burnet gave of such and “accidental” transfer was when a parasite that had evolved to become a saprophyte in the alimentary tract of a blood-sucking insect was accidentally transferred to man via an insect bite.

18 Burnet’s debt to Elton is particularly evident in *Biological Aspects* where he argues that microbiology could be considered a branch of “animal ecology” (Burnet 1940, p. 4).

19 Meyer’s paper appeared in the same Harvey Society volume as a paper by Dubos on the utilization of microbes in the study of biological problems (Dubos 1939–1940). Dubos most likely became aware of Meyer’s research while devising an aeration column for the production of large quantities of Shiga bacillus toxin for the United States biological weapons programme—research that prompted Meyer to write to E. B. Fred, who was coordinating research on potential biowarfare agents, to request that the Harvard researcher forward instructions for the column to the Hooper Foundation so that he could use it in his work on plague. Thereafter, Meyer referred to his Harvard and Rockefeller colleague as “Brother Dubos”. Karl Meyer Papers, Bancroft Library, Berkeley. BANC 76/42cz, Box 11, “General correspondence—plague project work.”

20 Burnet to Linda Burnet, 16 January 1944. Frank Macfarlane Burnet Papers, Baillieu Library, University of Melbourne.

21 For a summary of Dochez and Avery’s investigations see Dubos (1976c, pp. 103–108, 116–123).

22 Scientific staff biographical files, Rockefeller University Records, FA260, Box 10, Folder 27. “Fenner, Frank J., 1948–1949.”

23 F. Fenner, “A lateral arabesque—from virology to environmental science.” Unpublished lecture, 1974, p. 4. Frank Fenner Papers, Basser Library, Australian Academy of Sciences, Canberra. Box 9, Folder 11/4B “Lectures and Unpublished Articles, 1974.”

24 Stephen Boyden, information to author, 16 March 2017.

25 WHO, “BCG Vaccine.” http://www.who.int/biologicals/areas/vaccines/bcg/en/. Accessed 6 May 2017.

26 Dubos’s insights are supported by modern TB researchers who recognise that the caseating granuloma is the characteristic lesion of all TB infections and that immunity is mediated by macrophages and T cells that enhance granuloma function. However, scientists are still unable to explain why immunity to infection does not enable susceptible humans to resolve lung function and prevent development of the disease (Hunter 2016).

27 R. Dubos, “Infection in ‘disease free’ animals.” Health Research Grants and Annual Reports, The Rockefeller Institute, 1962. René Dubos Papers, Rockefeller University Archives, RU 450 D851, Box 54, Folder 1.

28 The phrase appears in a letter from Frank and Bobbie Fenner to Dubos on 21 December 1970. “Biographical Material, 1975–1981.” René Dubos Papers, Rockefeller University Archives, RU 450 DA51, Box 1, Folder 2.

29 Dubos to Fenner, 29 March 1951. Frank Fenner Papers, Basser Library, Australian Academy of Sciences, Canberra. MS 143, Box 3, Folder 7 “Rockefeller Institute of Medical Research.”

30 Dubos to Fenner, 10 May 1950. Frank Fenner Papers, Basser Library, Australian Academy of Sciences, Canberra. MS 143, Box 3, Folder 7 “Rockefeller Institute of Medical Research.”

31 Fenner to Dubos, 10 July 1950. Frank Fenner Papers, Basser Library, Australian Academy of Sciences, Canberra. MS 143, Box 3, Folder 7 “Rockefeller Institute of Medical Research.”

32 R. Dubos, “Infection into disease”. Paper for conference on psittacosis at Rockefeller Institute, New York, March 1954, pp. 2–4. René Dubos Papers, Rockefeller University Archives. RU450 D851, Box 26, Folder 6. Dubos most likely owed this insight to Meyer, who, together with Burnet, made a close study of the ecology of psittacosis in the 1930s and who summarized his findings in a 1942 De Lamaar lecture (Meyer 1942).

33 For further discussion of Dubos’s research in this period and his contribution to ideas of the microbiome see Sangodeyi (2012).
